# Preemptive Impella 5.5 insertion to reduce operative risk in high-risk cardiac surgery: A case report

**DOI:** 10.1016/j.ijscr.2024.109947

**Published:** 2024-06-27

**Authors:** Chidiebere Peter Echieh, Alex Ryan, Abel Cherian, Yash Rohilla, Kevin Wang, Toshinobu Kazui

**Affiliations:** a Division of Cardiothoracic Surgery, Department of Surgery, University of Arizona, United States of America; b University of Arizona College of Medicine, United States of America; c University of Arizona College of Science, United States of America; d Department of Surgery, Banner University Medical Center Tucson, United States of America; e Division of Cardiothoracic Surgery, Department of Surgery, Banner University Medical Center, Tucson, United States of America

**Keywords:** Impella 5.5, High-risk cardiac surgery, Mechanical circulatory support, Post cardiotomy shock, Concomitant implantation of Impella 5.5

## Abstract

**Introduction and importance:**

Society of thoracic surgery (STS) risk score has been used as a tool to gauge operative risk of cardiac surgery patients. High-risk patients, with STS risk score > 8 %, are considered as having prohibitive risk and are not offered surgery. There is no established strategy to minimize postoperative hemodynamic instability using mechanical circulatory support (MCS), despite growing interest in utilizing MCS prior to hemodynamic instability. The Impella 5.5 can provide enough perfusion and unload the left ventricle.

**Case presentation:**

We managed a 75-year-old male with multiple comorbidities and a presumed Society of Thoracic Surgeons (STS) score higher than 9.8 %, who had redo coronary artery bypass grafting and aortic and mitral valve replacement with concomitant implantation of the Impella 5.5. Patient had a good recovery despite developing post-operative atrial fibrillation.

**Discussion:**

Impella is used as a mechanical circulatory support device in patients with cardiogenic shock. It provides forward flow and effectively unloads the left ventricle. The concomitant placement of the Impella 5.5 in high-risk cardiac candidates may be associated with reduced operative risk.

**Conclusion:**

Placement of the device as part of surgical plan can potentially mitigate the perioperative risk by providing adequate endogean perfusion, decrease pressor support, unloading LV.

## Highlights

• Patients with high STS risk score are considered high risk and may not be offered surgery.
• Preemptive use of Impella may reduce the occurrence of post cardiotomy shock in high-risk cardiac surgery.
• Indications for preemptive implantation of Impella are not standardized.

## 1 Introduction

With increasing age and complexity of patients referred for cardiac surgery, a significant proportion of patients undergoing cardiac surgery are considered high risk with attendant uncertainty in outcome [1]. The Society of Thoracic Surgeons (STS) score is used to estimate the risk of cardiac surgery. Patients with an STS predicted risk of surgical mortality >8 % are considered high risk. In these patients, the development of low cardiac output syndrome in the early postoperative period, which manifests as post cardiotomy shock, is often the initial event that spirals down to multiorgan failure and poor outcome [2].

Presently, there is no optimal therapy to reduce the operative risk in these patients, which prevents high-risk patients from being offered surgical therapy in many cases. A potential solution is the use of mechanical circulatory support (MCS) in the perioperative period. An intra-aortic balloon pump (IABP) has been used as MCS in patients going for high-risk cardiac surgery with the intent to reduce surgical risk, however; studies have demonstrated contradictory results of IABP in high-risk patients [3]. Extracorporeal membrane oxygenation (ECMO) may be used; however, it is limited due to the requirement for anticoagulation, patient immobilization, and bleeding risk. [[4], [5], [6]] Micro-axillary pump, Impella 5.5® (Abiomed, Danvers, MA, USA), has been used as a potent MCS device perioperatively with multiple different purposes, such as preoperative optimization, bridge to recovery, perioperative circulatory support, and left ventricular venting. Currently, there is no consensus on the ideal modality for preemptive MCS or the timing of initiation of support in high-risk surgery. Furthermore, contrary to durable left ventricular assist devices (LVADs), temporary LVADs have not been comprehensively studied.

We present a case that underwent implantation of the Impella 5.5 during a high-risk surgery as part of a planned procedure that included redo coronary artery bypass grafting (CABG), mitral valve replacement, and aortic valve replacement. The patient was successfully supported during the postoperative period and recovered from the surgery without complications. This case report has been done in keeping with the updated SCARE guidelines [7].

## 2 Case report

**Fig. 1:**
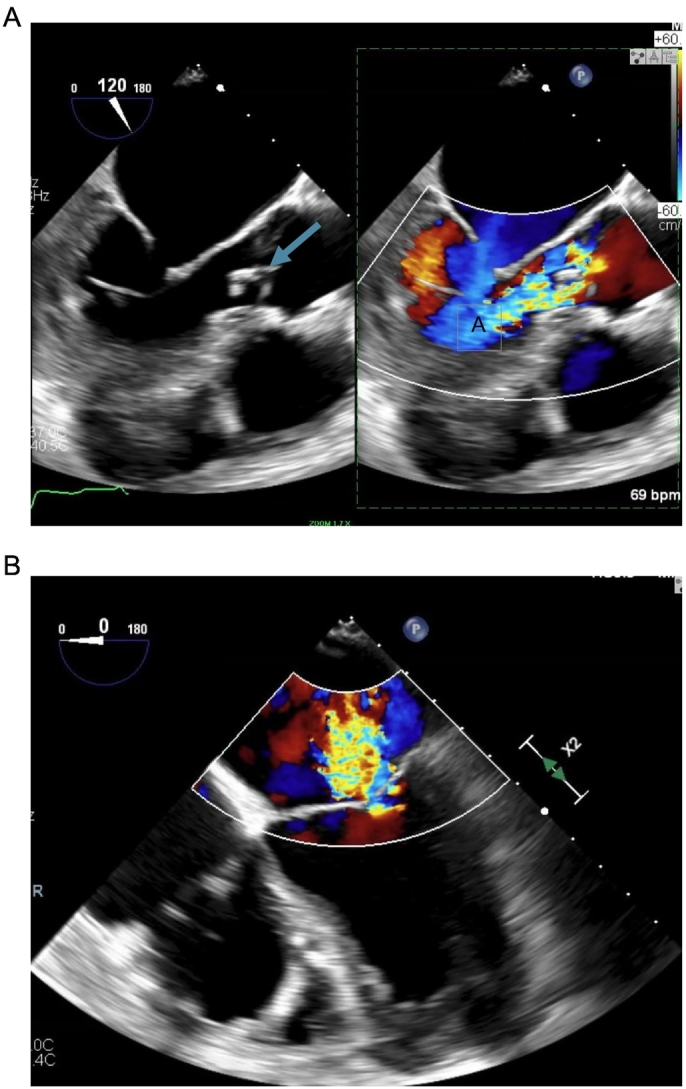
A: TEE showing aortic valve vegetation (blue arrow) and aortic regurgitation (A). B: TEE showing mitral valve regurgitation.

**Fig. 2:**
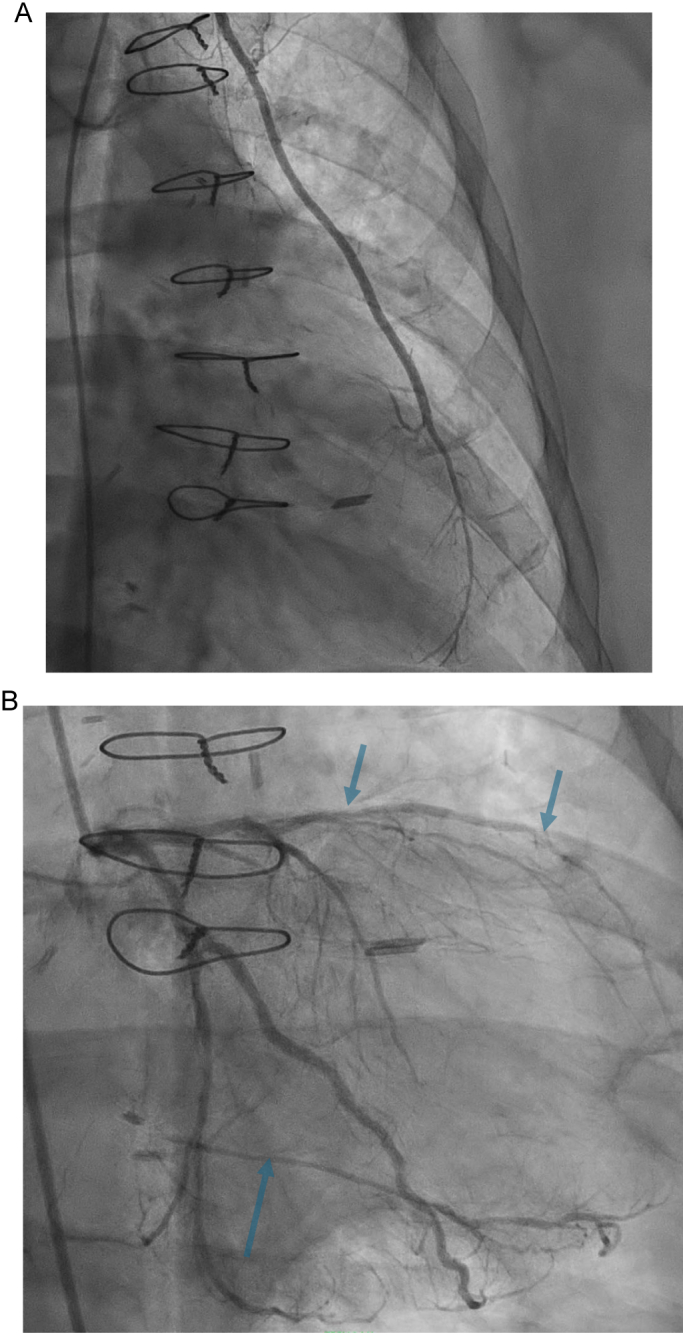
A: Angiography showing patent left internal thoracic artery to left anterior descending artery anastomosis. B: Showing coronary artery disease in LAD and left circumflex with retrograde filling of the right coronary artery.

We managed a 75-year-old male with chronic heart failure with a reduced left ventricular ejection fraction (LVEF) of 35 %. He underwent prior CABG in 2014, which included left internal mammary artery to left anterior descending artery (LAD), saphenous vein graft (SVG) to obtuse marginal branch (OM), 2nd diagonal branch (D2), and posterior descending artery (PDA). The patient then deteriorated from the New York Heart Association functional class I to III at nine years after the index operation, with progressively worsening dyspnea and chest pressure. Transesophageal echocardiography demonstrated a calcific aortic valve with 0.9 cm chronic, calcified vegetation on the non-coronary cusp, moderate aortic regurgitation of 3.4 cm^2^, and severe functional mitral regurgitation secondary to mitral leaflet mal-coaptation with mitral valve effective regurgitant orifice by Proximal Isovelocity Surface Area (PISA) being 72.6 mm^2^ (Fig. 1A and B). There was moderate left ventricular systolic dysfunction with an LVEF of 30–35 %, and the right ventricle was dilated with reduced contractility. A coronary angiogram showed patent left main, mid-segment occlusion of the left anterior descending (LAD) artery, patent left internal mammary artery (LIMA) to LAD anastomosis, diffusely diseased D2, non-dominant circumflex with midsegment 80 % stenosis, and midsegment occlusion of the right coronary artery (Fig. 2A and B) His average 5-meter walk time was 4.7 s.

Treatment options were discussed with the patient. Given there were not any appropriate percutaneous options, the patient consented to undergo aortic valve replacement (AVR), mitral valve replacement (MVR), redo CABG (SVG-PDA). In addition, implantation of the Impella 5.5 was planned to prevent postoperative low cardiac status after MVR with baseline reduced LVEF. Planned postoperative percutaneous coronary intervention (PCI) to OM was adopted to reduce the complexity of the surgery. The patient underwent elective redo-surgery. The redo-sternotomy was performed. The heart was dissected out and cardiopulmonary bypass was established via central cannulation. The right SVG was harvested with endoscope. Del Nido cardioplegia was administered in antegrade and retrograde fashion. A second dose was administered at 60 min in retrograde fashion. The PDA distal to the previous SVG was performed. MVR was performed via Sondergaard's groove. We placed a 31 mm Mitris mitral valve with the posterior leaflet preserved. Subsequently, AVR was performed with a 25 mm Inspiris valve placed in supra-annular position. For the Impella 5.5 implantation, a 10 mm Gelweave tube graft anastomosis was constructed to the distal ascending aorta in an end-to-side fashion and the graft was pulled out above the right clavicle. The Impella device was introduced through the aortic approach and a punch was utilized to create the tract for the Impella in the descending aorta. The Impella 5.5 sheath was placed to the end of the graft and the Impella device was advanced through the graft and placed through the Inspiris aortic valve. The position of the device was confirmed using transesophageal echo. Aortotomy was closed and cross clamp removed. Circulatory support with Impella 5.5 was instituted. Pump and clamp time were 216 and 154 min, respectively. Inotropic support in the postoperative period included norepinephrine, epinephrine, dobutamine, milrinone, vasopressin, and inhaled nitric oxide (30 ppm). He was extubated on postoperative day 2. Dobutamine was weaned on postoperative day 4. All inotropes were discontinued as congestions were resolved by day 7. Recovery was uneventful except for development of postoperative atrial fibrillation on postoperative day 5, which was treated with amiodarone with a successful conversion to sinus rhythm. MCS with Impella 5.5 was gradually weaned off and removed on postoperative day 7. Two weeks after surgery, the patient had PCI for planned revascularization and stenting of the left circumflex and obtuse marginal arteries. With satisfactory recovery, the patient was discharged on dual antiplatelet therapy. Postoperative follow up showed improved functional status.

## 3 Discussion

This case demonstrated that complex and high-risk surgery with reduced LVEF can be performed safely with preemptive Impella 5.5 placement at the time of surgery. High-risk cardiac surgical patients are identified by their advanced age, surgical history, severely reduced LVEF/congestive heart failure, presence of comorbidities, and complexity of the planned surgery. Preoperative risk scoring can be done using the STS risk score and frailty assessment. This helps inform shared decision making, preoperative planning, and preoperative optimization with modifiable risk factors. The STS calculator estimated the risk of morbidity and mortality for this patient to be 9.8 % for CABG plus MVR and 5.6 % for CABG plus AVR after medical optimization. This risk is significantly high and warrants the use of patient specific considerations such as preemptive use of MCS to prevent post cardiotomy shock, especially for this patient with severely depressed LVEF.

Utilization of several MCS devices in the postoperative period has been reported. In addition, use of the Impella 5.5 as part of a planned procedure has been reported to be successful in patients with cardiogenic shock. This case, however, highlights the use of Impella in high-risk cardiac surgery patients who are frail. This case illustrated that the Impella 5.5 provided much needed flow support in a patient who was deemed a high-risk surgical candidate who demonstrated pre-operatively that he may not tolerate the surgery given his lack of physiological reserve. Despite the use of Impella, patient required multimodal inotropic support in the post-operative period.

The benefit of the Impella 5.5 during cardiac surgery is to provide forward flow while adequately unloading the left ventricle; this prevents post-cardiotomy shock and minimizes the need for high dose inotropic and pressor support [3]. Avoiding high dose inotropic and pressors support in reduced LV function mitigates the risk of LV failure and end-organ damage postoperatively. We argue that, in this patient, high dose inotropic support in the absence of Impella would not have been adequate in the post-operative period. Furthermore, Impella® 5.5 has the advantage of providing perfusion in an ambulatory capacity [8]. There is the possibility that use of Impella® across a bio-prosthetic valve may cause precipitous bioprosthetic valve degeneration. However, examination of explanted hearts in heart transplant patients who had extended use of Impella® as bridge to transplant, did not show features of aortic valve injury [9]. While it is possible that the bioprosthetic valve material may respond differently to the foreign body across the valve, we did not see any echocardiographic feature of valve degeneration in our case.

Experience from the elective use of other support devices such as IABP for PCI did not show significant reduction in the occurrence of major adverse cardiovascular and cerebral events (MACCEs) [10]; however, recent guidelines recommend use of IABP in post-cardiotomy shock [11,12]. Currently, use of the Impella 5.5 in patients is mostly post hoc as bridge to recovery or bridge to transplant in patients with postoperative complications [13]. There is an opinion that the Impella 5.5 has been used for preemptive ventricular unloading and that prophylactic use of the Impella device to limit left ventricular dysfunction during and following high-risk cardiac operations would be a new use of this device [14].

Available data from the PROTECT II study, showed lower MACCEs in the Impella arm at 90 days [15]. Additionally, a recent report of successful implantation of Impella 5.5 under local anesthesia provides increased versatility, allowing preoperative institution of circulatory support prior to induction of general anesthesia in frail high-risk patients [16].

There are some limitations with the Impella device. It is associated with increased incidence of vascular complications and limb ischemia [17]. Insertion of the Impella 5.5 adds complexity and risk to the surgery. In addition, there is not clear evidence to decide which patients should get this therapy at the time of planned surgery. The preemptive use of the Impella device to prophylactically reduce the left ventricular work in high-risk patients is a new and appealing idea that needs to be backed by evidence. The evidence to support this use may only be obtained by experiential observations and anecdotal reports [14]. The Impella Protected Cardiac Surgery Trial is intended to test the feasibility of enrolment for clinical trial. This case report documents successful preemptive use and provides anecdotal evidence that may inform clinical application.

## 4 Conclusion

Placement of the Impella 5.5 as part of planned procedure for complex cases with reduced LVEF can be a good option to prevent post-cardiotomy shock. Further study is mandatory to identify the right patient population for this approach.

## Patient perspective

Patient was satisfied with the outcome of care received.

## Abbreviations

CABG: Coronary Artery Bypass Grafting
D2: Second Diagonal Artery
ECMO: Extra-Corporeal Membrane Oxygenation
LAD: Left Anterior Descending Artery
LVEF: Left Ventricular Ejection Fraction
LIMA: Left Internal Mammary Artery
LVAD: Left Ventricular Assist Device
MACCEs: Major Adverse Cardiovascular and Cerebral Events
MCS: Mechanical Circulatory Support
OM: Obtuse Marginal artery
PCI: Percutaneous Coronary Intervention
PDA: Posterior Descending Artery
RCA: Right Coronary Artery
STS: Society of Thoracic Surgeons
SVG: Saphenous Vein Graft

## Ethical approval

Ethical approval for the publication of this case report was obtained from the Ethical Committee/Privacy Unit of the Banner University Medical Center, University of Arizona on October 26, 2023.

## Funding

None

## Author contribution

Chidiebere Peter Echieh, FRCS: Reviewed and approved the manuscript.

Alex Ryan, BS^2^: Reviewed and approved the manuscript.

Abel Cherian, BS^2^: Reviewed and approved the manuscript.

Yash Rohilla^3^: Wrote and approved the manuscript.

Kevin Wang, MD^4^: Reviewed and approved the manuscript.

Toshinobu Kazui, MD, PhD: Performed the surgery, reviewed and approved the manuscript.

## Guarantor

Chidiebere Peter Echieh, FRCS

## Research registration number

This case report was not registered.

It is neither a clinical trial nor was reported as first in man.

## Consent

Written informed consent was obtained from the patient for publication of this case report and accompanying images. A copy of the written consent is available for review by the Editor-in-Chief of this journal on request.

## Conflict of interest statement

None.

## References

[1] Westaby S., Balacumaraswami L., Sayeed R. (2007). Maximizing survival potential in very high risk cardiac surgery. Heart Fail. Clin..

[2] Imazio M., Brucato A., Rovere M.E., Gandino A., Cemin R., Ferrua S. (2011). Contemporary features, risk factors, and prognosis of the post-pericardiotomy syndrome. Am. J. Cardiol..

[3] Metz D., Stiller M., Silber R.E., Kroll H., Hofmann H.S., Diez C. (2011). Prophylactic intraaortic balloon pumping in high-risk cardiac surgery patients. Med Klin Intensivmed Notfmed..

[4] Stefan M., Stiru O., Marinica I., Luchian M., Paunescu A., Ciurciun A. (2020). Extracorporeal membrane oxygenation (ECMO) rescue therapy in post-CARDIOTOMY cardiogenic shock: a CASE report. Romanian Journal of Anesthaesia and Intensive Care..

[5] Lorusso R., Kowalewski M., Di Mauro M., Mariani S. (2022). After the storm comes a calm: the (rather good) post-discharge survival of adults undergoing post-cardiotomy extracorporeal life support. Eur. J. Cardiothorac. Surg..

[6] Kumar T.K.S., Zurakowski D., Dalton H., Talwar S., Allard-Picou A., Duebener L.F. (2010). Extracorporeal membrane oxygenation in postcardiotomy patients: factors influencing outcome. Journal of Thoracic and Cardiovascular Surgery..

[7] Sohrabi C., Mathew G., Maria N., Kerwan A., Franchi T., Agha R.A. (2023). The SCARE 2023 guideline: updating consensus surgical CAse REport (SCARE) guidelines. Int. J. Surg..

[8] Tsuneyoshi H, Rao V. The Role of Extracorporeal Membrane Oxygenation (ECMO) Therapy in Acute Heart Failure. www.anesthesiaclinics.com. 10.1097/AIA.0b013e3182603ed5 22735722

[9] Pepe R.J., Soliman F.K., Grewal J., Dulnuan K., Huang M., Iyer D. (2024). Dilemma at low-volume heart transplant center provides the potential for extended Impella 5.5 use as a bridge to heart transplant. J. Heart Lung Transplant..

[10] Zeymer U., Bauer T., Hamm C., Zahn R., Weidinger F., Seabra-Gomes R. (2011). Use and impact of intra-aortic balloon pump on mortality in patients with acute myocardial infarction complicated by cardiogenic shock: results of the euro heart survey on PCI. EuroIntervention.

[11] Lorusso R., Whitman G., Milojevic M., Raffa G., Mcmullan D.M., Boeken U. (2021). 2020 EACTS/ELSO/STS/AATS expert consensus on post-cardiotomy extracorporeal life support in adult patients. Eur. J. Cardiothorac. Surg..

[12] Kirklin J.K., Pagani F.D., Goldstein D.J., John R., Rogers J.G., Atluri P. (2020). American Association for Thoracic Surgery/International Society for Heart and Lung Transplantation guidelines on selected topics in mechanical circulatory support. J. Thorac. Cardiovasc. Surg..

[13] Newman J.S., Demekhin V., Piper G.L., Tohme S., Haddad O., Kon Z. (2024). Heparin free zone: 100 days of Impella 5.5 support with Argatroban in a patient with HIT. J. Heart Lung Transplant..

[14] Ferraris V.A. (2021). Preemptive left ventricular unloading in high-risk cardiac operations—uncertain risk/benefit relationships, but promising!. JTCVS Open..

[15] O’Neill W.W., Kleiman N.S., Moses J., Henriques J.P., Dixon S., Massaro J. (2012). A prospective, randomized clinical trial of hemodynamic support with Impella 2.5 versus intra-aortic balloon pump in patients undergoing high-risk percutaneous coronary intervention the PROTECT II study. Circulation.

[16] Kaveh Eghbalzadeh, Wahlers TCW, Deppe AC. Implanting Impella 5.5 under local anaesthesia. *Thorac Cardiovasc Surg*. Published online 2023. DOI 10.1055/a-2132-4694. Accessed September 19, 2023. 37463601

[17] Philipson D.J., Cohen D.J., Fonarow G.C., Ziaeian B. (2021). Analysis of adverse events related to Impella usage (from the manufacturer and user facility device experience and National Inpatient Sample Databases). American Journal of Cardiology..

